# Validation of road traffic noise and prediction of traffic increases along a highway in Hungary using noise maps

**DOI:** 10.1007/s10661-026-15821-0

**Published:** 2026-08-24

**Authors:** Karam Waleed Katw, Bela Tóthmérész, Denes Kocsis

**Affiliations:** 1 Doctoral School of Environmental Science, University of Debrecen, 4032 Debrecen Egyetem tér 1, Debrecen, Hungary; 2 Department of Ecology, Faculty of Science and Technology, University of Debrecen, 4032 Debrecen Egyetem tér 1, Debrecen, Hungary; 3 Department of Environmental Engineering, Faculty of Engineering, University of Debrecen, 4032 Debrecen Egyetem tér 1, Debrecen, Hungary

**Keywords:** CNOSSOS-EU method, Noise barrier, Noise modelling, Model validation, Human population exposure

## Abstract

In urban regions of Europe, road traffic noise ranks as the second most significant environmental stressor, following air pollution. This study measures urban road traffic noise using a noise map that is validated by in situ noise measurements, and it predicts a future scenario for increased traffic in 2030 by altering the traffic composition and overall vehicle count whilst evaluating the noise exposure at building façade points. The investigation used the noise mapping software IMMI and employed the CNOSSOS-EU method to assess road traffic noise. There are strong connections between the actual noise measurements and the model results, with an average difference of 1.09 dB(A) on the roadside, whilst on the receptor side it was 0.81 dB(A). Urban transport development provides the basic data to predict the noise levels affecting the designated area. Based on the 35% increase expected in average day and night traffic flow in 2030, the results indicate an additional 307.14% of people will be exposed to noise levels between 55 and 60 dB(A) throughout the day (L_day_), and an additional 450.00% of people will be exposed to noise levels between 50 and 55 dB(A) at night (L_night_). By 2030, the continual increase in urban traffic, driven by population growth, will result in significantly heightened noise levels in densely populated areas. Furthermore, average day and night noise levels in high-traffic zones are anticipated to exceed 55 dB(A) without strategic urban planning and stringent enforcement of noise laws, which would exceed WHO health safety standards.

## Introduction

Noise has emerged as a major issue adversely impacting human health in today’s urban life, as urban planning techniques often fail to take environmental noise into account (Zannin et al., 2018). Environmental noise pollution refers to excessive or disruptive noise generated by human activities such as industry, transportation, and construction. A growing body of research has investigated the effects of transport noise and its association with annoyance (Khomenko et al., 2022; Minichilli et al., 2018), sleep disturbances (Halonen et al., 2012; Park et al., 2018), hypertension (Petri et al., 2021), stroke (Roswall et al., 2021), ischaemic heart disease (Vienneau et al., 2022), and metabolic syndrome (Li et al., 2021). Recent studies have looked into possible links between environmental noise and several health problems, such as breathing problems (Carey et al., 2016), obesity (Christensen et al., 2015; Veber et al., 2023), diabetes (Ashin et al., 2018; Wang et al., 2020), problems with the immune system (Kim et al., 2017), mental health issues and stress (Thompson et al., 2022; Vukić et al., 2021), and problems with childhood and foetal development (Gupta et al., 2018). Recent research suggests a possible connection between cancer and environmental noise (Hegewald et al., 2017; Thacher et al., 2023). Researchers estimate that each year in Europe, environmental noise results in the loss of over one million healthy years of life (Rodrigues, 2018).

In Europe, road traffic noise ranks as the second most common hazardous factor to human well-being, after fine particle pollution (Hänninen et al., 2014). In actuality, noise levels from road traffic which exceed established thresholds impact 25% of the population in Europe (European Environment Agency, 2017). An exponential increase in the number of vehicles worldwide leads to a corresponding increase in traffic noise levels (Nedic et al., 2014). For instance, research suggests that exposure to long-term noise causes 12,000 preventable deaths annually in Europe (European Environment Agency, 2023). It is projected that the total population subjected to L_den_ ≥ 55 dB is approximately 113 million due to road traffic noise, 22 million from railway noise, 4 million from aviation noise, and 1 million from industrial noise, whilst 79 million individuals are subjected to L_night_ of no less than 50 decibels (A) as a result of road traffic (European Environment Agency,). Thus, vehicle traffic undoubtedly constitutes the largest source of noise in European cities, as well as other urban areas globally (Alam et al., 2020) and villages (Rey Gozalo et al., 2013). Regrettably, the current rate of implementing needed mitigation measures does not anticipate a substantial reduction by 2030 (Wilkki & Reeve, 2021). The Organisation for Economic Cooperation and Development (OECD) classifies urban areas with an L_den_ ≥ 65 dB as black acoustic zones (Alexandre & Barde, 1990). Europe is failing to achieve its main goal of drastically lowering noise pollution. Other nations have also observed a rise in road traffic noise. The International Passenger Transport Forum predicts that the demand for transport will nearly triple by 2050, particularly through private vehicles, due to population expansion and improving quality of life (International Transport Forum, 2019). Population expansion and urban traffic are among the potential factors that have offset efforts to decrease noise levels (European Environment Agency, 2019b; Rodrigue, 2020). The objective of the Zero Pollution Action Plan is to decrease the number of individuals chronically exposed to transport noise by 30% by 2030 (Martinsohn, 2022). Consequently, existing noise reduction strategies must be augmented to attain this decrease.

Acoustic barriers are recognised not only as impediments to sound but also as visual obstructions (Jiang & Kang, 2016). Milford et al. (Milford et al., 2014) examined the most effective noise reduction strategies, including façade insulation, noise barriers, quieter roads, and quieter cars, in terms of their impact on cost-effectiveness and nuisance. They asserted that source-based noise reduction is the most economical method for mitigating noise nuisance (Milford et al., 2014), (Ranpise & Tandel, 2022). Noise mitigation on facades, as well as the implementation of low-noise tyres and quiet road surfaces, can achieve reductions of up to 10 dB, whilst barriers can attenuate noise by 15–20 dB (Rey-Gozalo et al., 2022). Due to poor design and limited access to alternatives with more effective techniques, noise barriers have become a prevalent strategy for reducing noise, particularly vehicle noise. Most of the approaches adopted to decrease perceived noise levels are associated with insulating windows and building facades, which may not yield effective results in the summer (Ouis, 2001). Additionally, some research has investigated noise mitigation strategies for installing barriers that include vegetative barriers (Margaritis et al., 2018) and physical barriers (Kim et al., 2007). Generally, numerous studies indicate that a combination of strategies from various sources may yield synergy in mitigating traffic noise.

In 2002, the Environmental Noise Directive (END), also known as EC Directive 2002/49/EC (European Commission, 2002), was adopted into EU law. EU Member States (MS) have been legally required since 2007 to create strategic noise maps every five years for noise from cars, trains, airplanes, and industry within agglomerations with more than 250,000 residents, and from 2012 for agglomerations with more than 100,000 residents. In addition to urban areas, it is necessary to map roads with more than 3 million vehicle crossings annually, trains with over 30,000 passages annually, and airports with more than 50,000 movements annually. The END was established to safeguard the European population from the adverse health impacts caused by ambient noise, driven by an increasing body of research indicating that such noise might result in adverse health outcomes (Murphy & King, 2014). A standardised method for noise mapping has been established as one of the aims of the END: to enhance consistency in noise evaluations throughout European member states. This methodology is known as CNOSSOS-EU (Kephalopoulos et al., 2014). The CNOSSOS-EU report, released in 2012, proposes a standard methodology for assessing population exposure throughout the EU, incorporating a uniform strategy for designating receiver points to structures, primarily based on the German national technique (VBEB) with some modifications. VBEB allocates the building’s population uniformly among all receiver points surrounding the structure and assesses exposure in relation to noise levels along all façades. The CNOSSOS-EU methodology facilitates exposure estimation in scenarios where population data is either accessible or inaccessible for buildings in designated regions (Kephalopoulos et al., 2014).

It is essential to have a thorough understanding of current exposure patterns to mitigate the problems related to road traffic noise. A method for obtaining and displaying this information is to use noise maps, which enable the evaluation and quantification of noise exposure in specific areas of interest. A noise map is a graphical depiction of the geographic distribution of noise across a defined time frame in a particular area. It serves as an effective instrument for managing and controlling environmental noise as well as for urban planning, and it provides a theoretical foundation for developing environmental noise legislation. The conventional, model-based approaches for generating noise maps rely on the implementation of noise mechanism models to compute and derive noise maps (Lan et al., 2020; Zambon et al., 2017). Here is a succinct outline of the methodology for calculating traffic noise maps: (1) The generation of noise receivers is achieved by gridding the area; (2) the sound pressure level for each receiver is computed using traffic noise emission and propagation models; (3) the visualisation of the noise distribution is achieved by applying spatial interpolation techniques to the sound level data. The noise maps naturally depict the spread of traffic noise, facilitating the assessment of noise pollution. In some studies, the noise map for a specific area is created by using data from noise monitoring stations or data collected manually, and the evaluation of traffic noise pollution is based on the average sound levels during the day or night (Lagonigro et al., 2018), which is suitable for accurately assessing small areas. Noise maps are typically generated using predictive methodologies. Spatial interpolation methods in Geographic Information Systems (GIS), including nearest-neighbour, kriging, spline, and inverse distance weighting, are extensively employed to generate detailed environmental noise maps. Enda Murphy (Murphy & Douglas, 2018) examined the impact of various noise factors on population exposure assessments. Gaetano Licitra measured the noise level at the grid point nearest to buildings to estimate residents’ noise exposure (Licitra et al., 2012), considering diverse noise sources such as roads, railways, airports, and industries in the assessment of disturbances (Licitra et al., 2011). The advancement of noise mapping enhances the precision of noise pollution assessment.

The evaluation of traffic noise pollution should prioritise its effects on people, particularly the influencing variables and characteristics of the impacted populations. Therefore, this work evaluated urban road traffic noise by using noise mapping validated by in situ noise measurements. In this context, the in situ noise measurements were conducted at a road section in the Kismacs suburban district of Debrecen, where the noise barrier is located near the roadside. This study proposes the adoption of the emission model (CNOSSOS-EU) [44]. Meanwhile, the IMMI noise mapping programme collects the population’s distribution data. The Hungarian public roads management authority provided average daily traffic data (ADT) for the reference scenario (scenario 0). The prediction of the future scenario (scenario 1) for traffic increases in 2030 is done by altering the traffic composition and overall vehicle count and evaluating the noise exposure at building façade points, according to urban transport development plans basis data. Finally, the study compared the future scenario with the reference case and assessed how well the simulated noise control methods would work in the hypothetical circumstances.

## Materials and methods

### Methodological framework

**Fig. 1:**
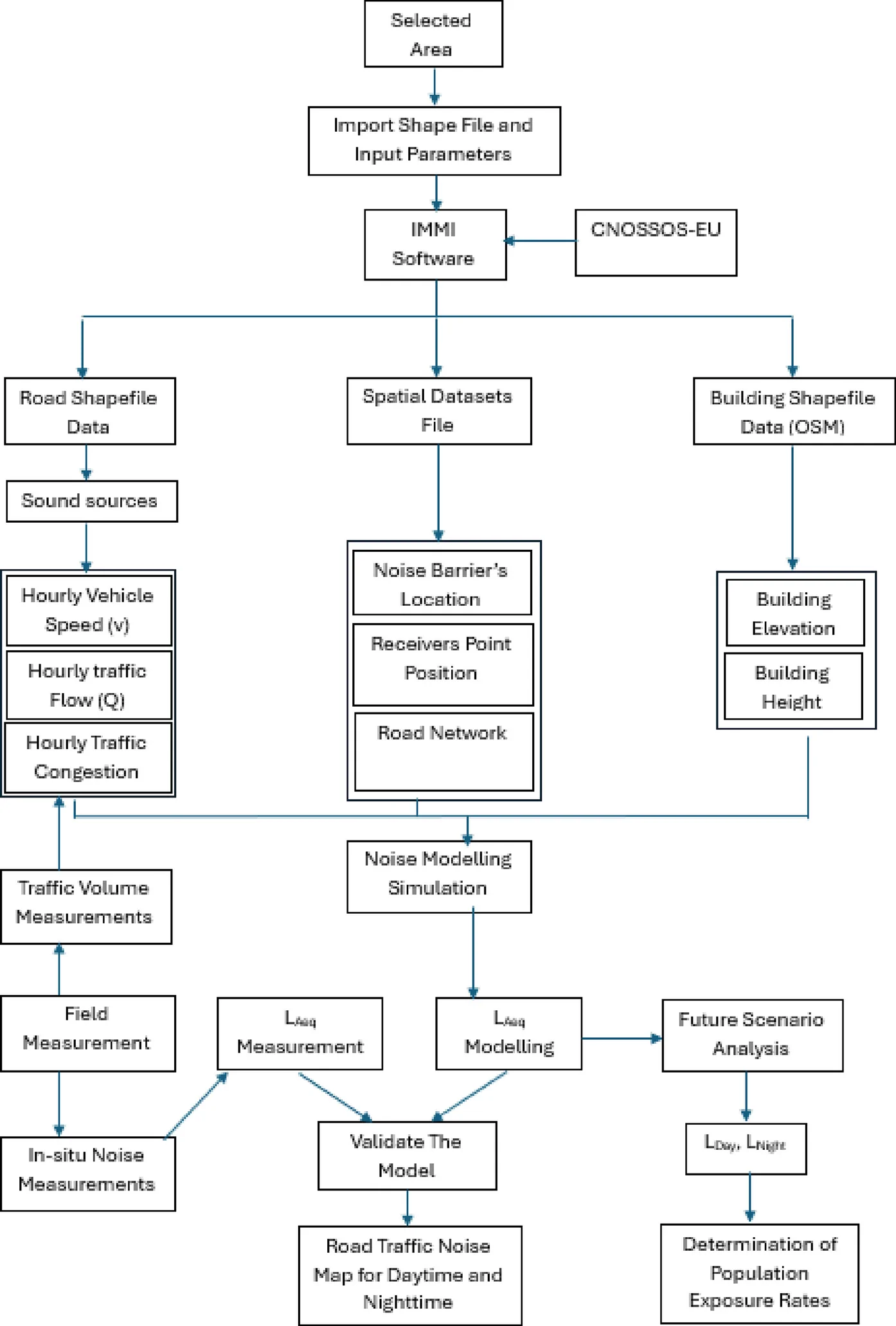
Methodological framework for the Kismacs study area

Figure 1 depicts the methodological framework. Using one of the city’s suburban areas as the study location, the in situ noise measurements were initially performed at the chosen measuring point simultaneously with the traffic volume measurements, including the type, position, and number of vehicles. Subsequently, the traffic noise across the chosen area was mapped using the IMMI software programme to validate the road traffic noise with the noise measurement. Finally, future scenario analysis is performed to assess the effects of traffic increases in 2030 and compare it with the reference case based on actual traffic data and building information.

### Study area

The analysis was conducted on the section of Hungarian main Road No. 33 that passes through the Kismacs suburban district of Debrecen. The city itself has experienced significant economic and population growth, resulting in negative effects on the transportation system, including traffic externalities. Road No. 33 is one of the busiest access roads to the city, and its load is expected to increase significantly with the opening of the northwest economic zone; therefore, it has been agreed to enlarge and update the impacted road segment. The investigated section is approximately 2 km long and features a dual carriageway with two lanes in each direction, which experiences significant traffic congestion and has highly mixed heterogeneous traffic. The southern part of the section is equipped with parallel rail trains in a two-way direction. The investigated section consists of three-legged intersections; at these intersections, traffic is controlled by traffic lights. Most of the buildings in the area are residential, ranging from one to two storeys with a maximum height of 6 m. The measurement sites were positioned at a suitable distance from the walls. Three intersection locations were selected along the Kismacs highway to evaluate noise pollution and count various types of vehicles for this study.

### Noise measurements

Given that vehicle traffic constitutes the primary source of environmental noise in urban environments (Montes González et al., 2023), the in situ investigation took place in a road section location near a noise barrier (the traffic crossroads that intersect the barrier divided the research area into three sections). The approximately 1-km-long and 4-m-high barrier is situated on the northern side of Road No. 33 in Debrecen. A series of outdoor noise measurements were conducted, each lasting 15 min, under suitable meteorological conditions (European Commission, 2002). The 15-min duration ensured that significant changes in noise levels were captured, even on low-traffic urban roads (ISO 1996−2, 2007).

**Fig. 2:**
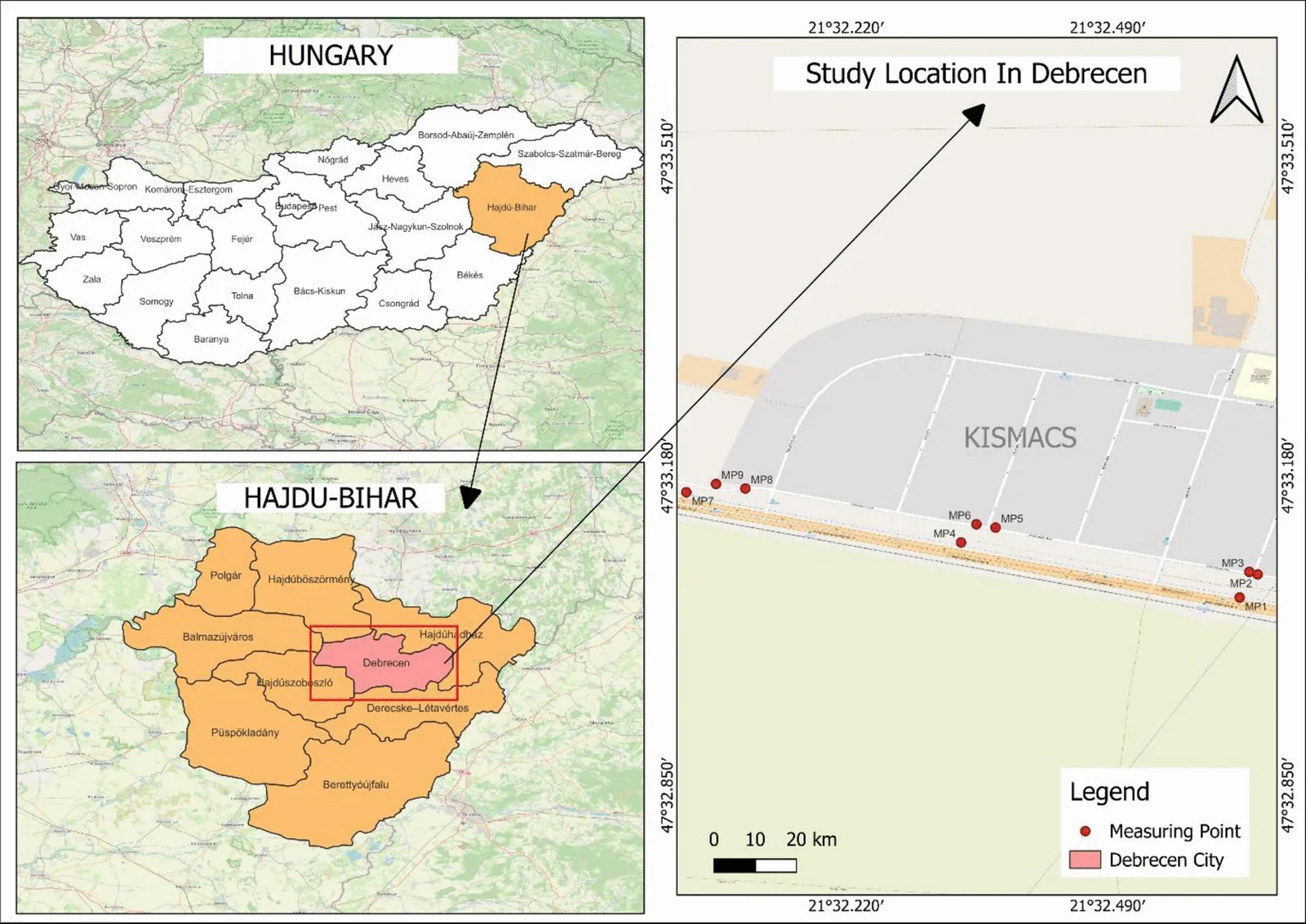
Measurement points (MP) for each location, along with the site design

There were two type 1 sound level meters (Fig. 2), which were placed near buildings behind the barrier, and a monitoring station, which was positioned horizontally 7.5 m away from the midpoint of the outer lane. They performed measurements synchronously on both the receptor and roadside of the barrier inside the research area to compare the noise measurements with the noise values generated by the model. The primary goal was to verify the coherence of the model computations for three specific points before moving on to other locations within the research region.

At every established measurement point, a set of four measurements was carried out. Every measurement had a duration of 15 min, with no breaks between measurements, resulting in a total timing of 1 h for each set. The set was repeated three times each day at the same measuring point. The measurement was conducted on three separate days—1 day for each barrier segment. After each 15-min measurement, the L_Aeq_ was computed, and an average hourly L_Aeq_ was obtained in relation to the four measurements. The L_Aeq_ value (in dB) was determined using Eq. (1), where L_Aeq_ represents each sound pressure level reading in dB and *n* represents the total number of measurements:

$${L}_{\mathrm{A}\mathrm{e}\mathrm{q}}=10\mathrm{log}\left(\frac{\sum\limits_{i=1}^{n}{10}^{0.1L\mathrm{A}\mathrm{e}\mathrm{q},i}}{n}\right)$$

For the analysis, the daytime rush hours on Tuesday, Friday, and Thursday, between 12:00 p.m. and 5:00 p.m. were chosen due to the largest hourly traffic volume during this period (which corresponds to the worst noise scenarios), in accordance with measurements made in European cities (Brambilla et al., 2023).

### Traffic volume measurements

Accurate traffic data is crucial for noise mapping to compute differences in road emissions based on the standards used. Within the IMMI programme, the emission level is calculated based on various influencing factors, including the number of vehicles in each vehicle category, road surface type, speed, and others. The input parameters can automatically determine the emission level. All roadway segments document the variations in emissions resulting from alterations in road surface, traffic speed, traffic volume, etc.

Measurements of traffic volume were performed by a manual vehicle count on the receptor and roadsides of the barrier for the vehicle classes defined in CNOSSOS-EU (Kephalopoulos et al., 2014): Category (Cat.) 1, Cat. 2, Cat. 3, Cat. 4a, and Cat. 4b. Road traffic counts were carried out continuously on both sides of the road for each different location. As previously stated, road traffic counts were carried out on the same day and at the same times as the in situ noise measurements of equivalent sound levels: during the late afternoon rush hours from 12:00 pm to 5:00 pm (“day” period). In the process of measuring traffic volume using manual counting, Road No. 33 and the adjacent streets were divided into two main lanes, each lane containing two or three sub-lanes that had varying traffic counts, as depicted in Fig. 2. The traffic counts have been recorded individually for each traffic direction. Table 2 displays statistics regarding vehicle counts.

### Noise mapping

Acoustic maps are highly valuable instruments for the diagnosis and assessment of levels of noise in urban areas (Zannin et al., 2013). Noise maps for a specific region are computed using various physical, geometrical, acoustic, and traffic factors. Sound propagation is an intricate phenomenon influenced by several elements. In an urban setting, the street layout, ground surface, buildings, and meteorological conditions have a significant influence. The CNOSSOS model delineates the calculation of sound trajectories, incorporating the influence of sound barriers and reflections from structures. However, calculating every possible trajectory is a complex process that requires an extensive understanding of the area’s topology. To achieve this objective, an acoustic map has been created using specialist acoustic analysis software. The analysis identifies areas of conflict, specifically those where road traffic noise exceeds the END reporting thresholds, such as L_den_ over 55 dB and L_night_ over 50 dB (European Environment Agency, 2018).

For the base map, Road No. 33 is designated as a linear noise source in the computational model. The source line was situated centrally in the roadway direction, utilising the “CNOSSOS-EU” calculation procedures. Road geometry, shapefiles, and building polygons near the measurement points obtained from the Debrecen urban development plan (which includes height data for structures) were separately modelled.

**Fig. 3:**
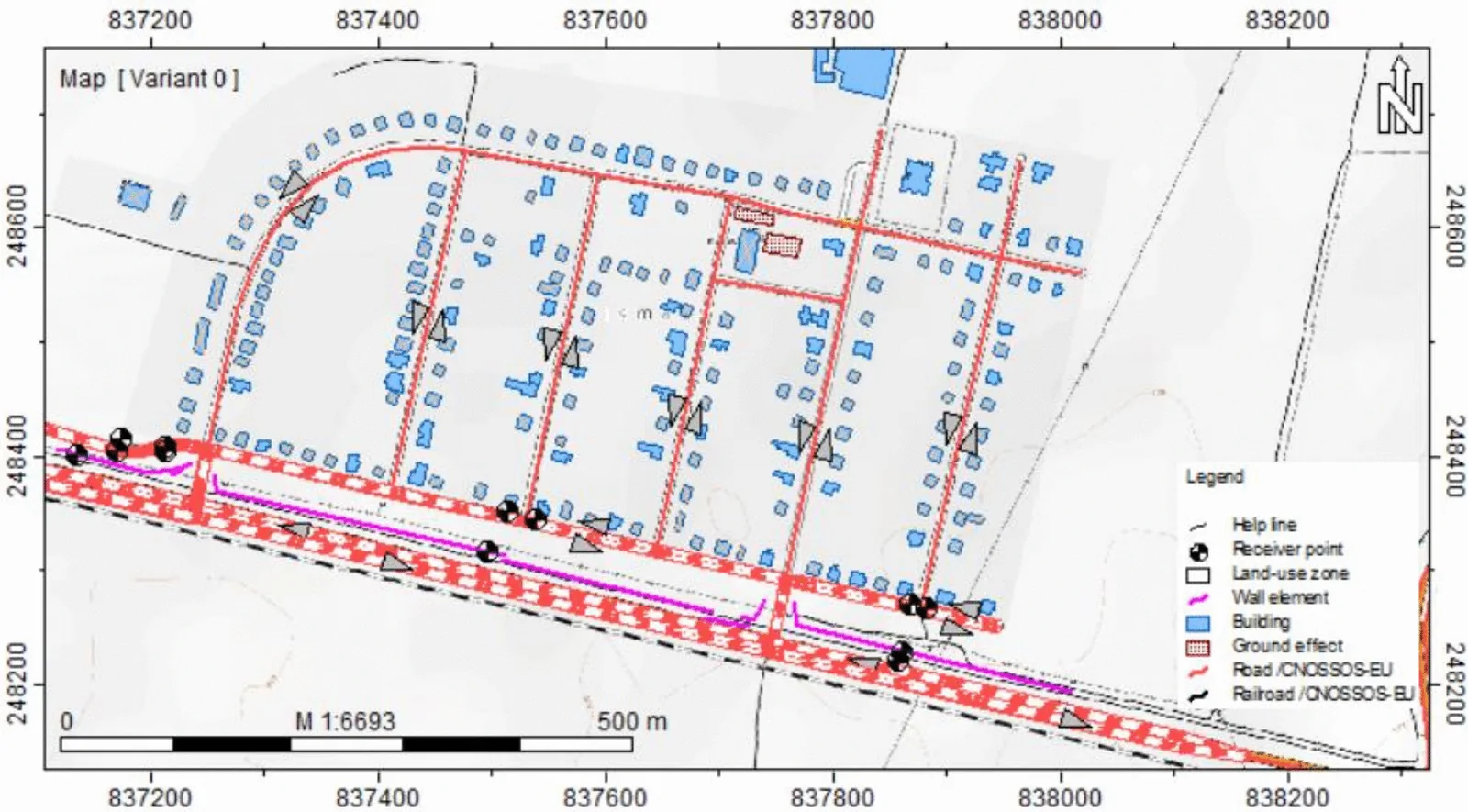
A cartographic representation of the research area under investigation

Environmental noise modelling was carried out using IMMI software (Wölfel Group, 2023), a dedicated acoustic simulation software widely used for environmental noise assessment and strategic noise mapping. IMMI simulates noise propagation from various sources and predicts the sound pressure levels, considers the building geometry, the atmospheric absorption, the reflections, and the meteorological conditions depending on the chosen calculation standard. The software integrates GIS-based spatial data to build high-resolution noise contour maps and to estimate the noise exposure at specific receptor locations. Figure 3 presents the aerial view of the base map for the subject area under examination.

#### Model validation

The validation technique aimed to reduce the discrepancy between the value obtained from the computational model and the measured sound level. In this work, the CNOSSOS-EU approach was used in the IMMI software to model noise and assess L_Aeq_ values based on manual traffic volume measurements. A meticulous validation of specific parameters is essential to compute the propagation of sound from the source to the receivers. During the validation procedure, adjustments were made to parameters exhibiting the greatest uncertainty, such as those pertaining to the average traffic speeds. The validation commenced when the comprehensive data had been input into the computer model, encompassing the following: comprehensive geometry of the distinct road segments, traffic volume, vehicle speed limits, as well as the type of pavement—determined through visual inspection, the geometry of shielding, attenuation, and the topographic model of the region. The IMMI noise mapping software was used to model noise sources identified as road segments characterised by hourly flow rates (Q) and specific average speeds (v) for every acoustic vehicle category under the assumption that traffic noise was the only sound source. Executing individual model runs for each measurement point, using “hourly distribution data” as input, was demonstrated to be the most suitable method for analysing the results. The allowable average traffic speeds were established based on the vertical sign inventory supplied by the Hungarian public road authority. The pavement type and condition in the model calculation were based on the real conditions seen during site visits for field measurements of noise levels.

OpenStreetMap is used to source the road and building network (Rabus et al., 2003). In the noise model, the receiver points were positioned at the same location as the in situ measurement. To achieve this goal, the GPS coordinate measurement device was used during the sample period. Calculation methods were used to estimate a total of 108 in situ 15-min noise measurement sessions. The reference surface in the model was determined to be the road surface’s characteristic, and the traffic flow for the research region was determined to be a continuous fluid flow. Most of the building heights are 3 m for the ground and 2 m for the roof.

#### Population exposure

An essential component of strategic noise mapping is the analysis of possible noise exposure among residents and its related detrimental impacts. As per section 2.8 of Annex II of Directive 2002/49/EC, the assessment entails quantifying noise emissions at the façades of residential structures [42]. Consequently, by determining the number of residences in a specified area, the assessment can calculate how many residents are subjected to noise. For this reason, the recorded average daily traffic (ADT) data provided by Hungarian Public Roads has been used for the reference scenario in 2022 (Table 1). Information regarding road network distribution, traffic intensity, road width, and speed limits was sourced from Hungarian Public Roads, reflecting the traffic conditions of 2022. The scenarios encompassed the locations and heights of buildings (both residential and non-residential) and vegetation, as well as the positions of roads and noise barriers (Fig. 3). Information on the 427 residents living in the area and their spatial distribution was obtained from the software tool. The raw traffic count data was sorted and organised into acoustic categories to produce the thorough traffic data required for the noise maps. The hourly vehicle traffic data for each lane were aggregated and subsequently merged into two time periods: day (06:00–18:00 h) and night (22:00–06:00 h) for both directions of the road. These time intervals correspond to the Hungarian legislation adopted in this study. Although the Environmental Noise Directive (END) defines day, evening, and night periods for the calculation of L_den_, the present study evaluates only L_day_ and L_night_ because these are the indicators required by the applicable national assessment framework and available traffic dataset.

**Table 1:** The recorded average daily traffic (ADT) data for the research area, categorised by acoustic vehicles (unit: vehicle/hour), with D indicating day and N indicating night

Time	Cat. 1	Cat. 2	Cat. 3	Cat. 4a	Cat. 4b
2022 D	770.58	12.70	27.27	1.55	1.55
2030 D prediction	1041.50	17.16	36.85	2.10	2.10
2022 N	148.71	2.73	6.47	0.34	0.34
2030 N prediction	200.99	3.69	8.74	0.45	0.45
Composition ratio	94.71%	1.56%	3.35%	0.19%	0.19%

Using software, the traffic prediction results are used to determine the noise emissions of the vehicles (L_day_ and L_night_) and the corresponding emissions at the receivers. To quantify the impact of the expected traffic flow increases in 2030 on road traffic noise, the noise map’s results were compared to the values determined by the same model but with the new traffic flow. Despite having varying traffic flow, all other input parameters and topographical requirements for the two maps were the same and were computed using the IMMI model. In this study, L_day_ and L_night_ noise key indicators for the prediction values were calculated based on a grid network of 20 m × 20 m in the research area. The noise maps are generated for the busiest Road No. 33, incorporating only the principal roads. Therefore, the actual variation in noise values may exceed that observed in the simulations.

## Results and discussion

### Analysis of observed traffic volume

Traffic information, including composition, hourly vehicle counts for the No. 33 highway road segment, and average vehicle speed, was collected. The data was arranged by measurement date, vehicle type, and traffic direction. Table 2 presents the traffic characteristics, including flow rate per direction.

**Table 2:** The manual count results for days 1, 2, and 3 consecutively

Traffic volume (veh/h)							
	Category	12:00 pm–1:00 pm		2:00 pm–3:00 pm		4:00 pm–5:00 pm	
		Westbound	Eastbound	Westbound	Eastbound	Westbound	Eastbound
Day 1	Cat. 1	282	268	333	267	460	348
	Cat. 2	24	28	27	21	29	25
	Cat. 3	16	18	11	16	12	13
	Cat. 4a	2	0	2	5	1	1
	Cat. 4b	0	4	0	0	1	0
Day 2	Category	3:15 pm–4:15 pm		4:30 pm–5:30 pm		5:45 pm–6:45 pm	
		Westbound	Eastbound	Westbound	Eastbound	Westbound	Eastbound
	Cat. 1	387	360	393	323	257	229
	Cat. 2	34	21	22	16	8	9
	Cat. 3	11	24	11	12	6	0
	Cat 4a	5	1	1	0	0	0
	Cat. 4b	0	1	1	0	0	0
Day 3	Category	2:00 pm–3:00 pm		3:15 pm–4:15 pm		4:30 pm–5:30 pm	
		Westbound	Eastbound	Westbound	Eastbound	Westbound	Eastbound
	Cat. 1	411	331	397	378	420	387
	Cat. 2	20	22	24	21	29	18
	Cat. 3	9	25	14	15	8	17
	Cat. 4a	2	4	4	1	0	4
	Cat. 4b	0	1	0	0	0	0

### Analysis of noise measurements

**Fig. 4:**
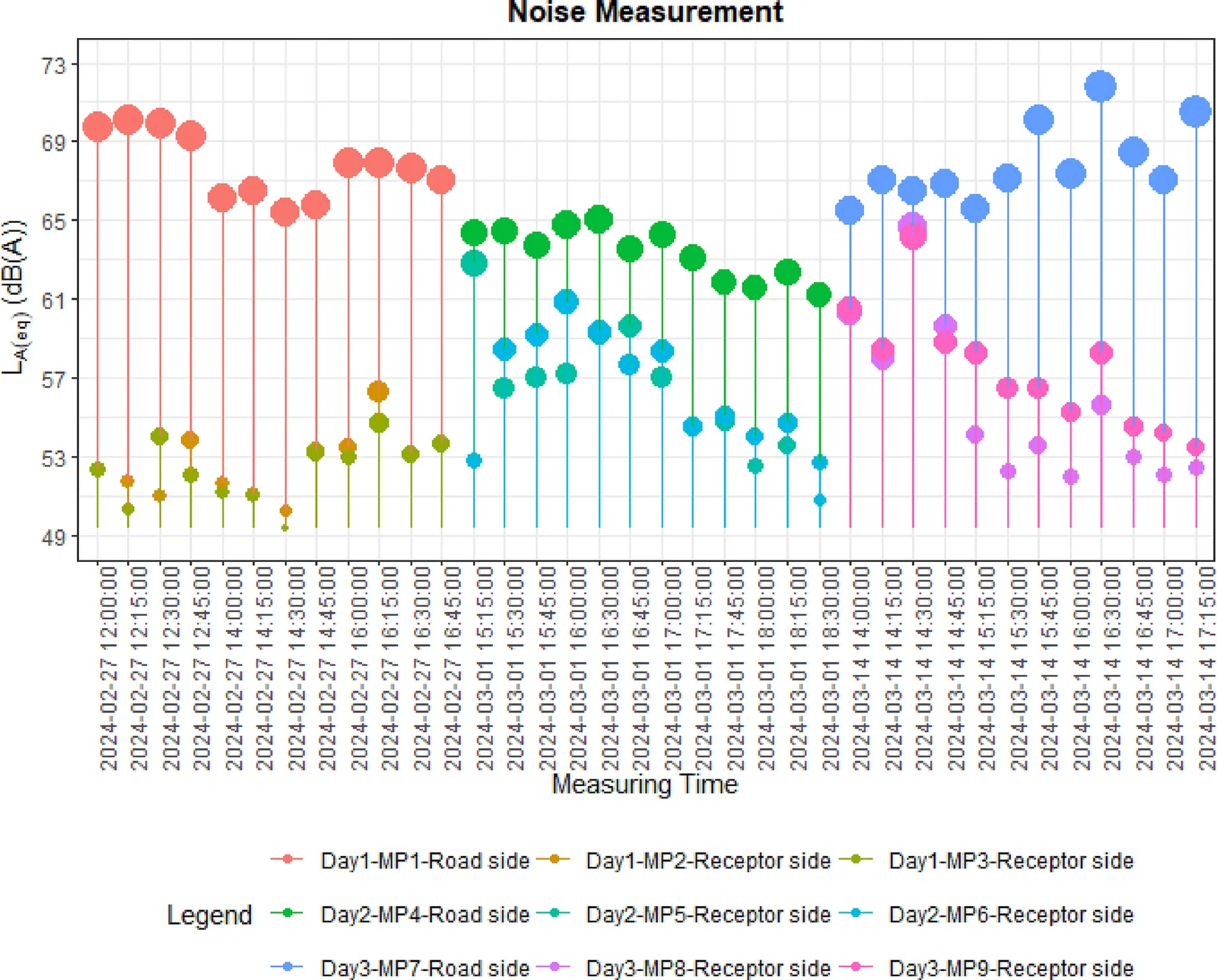
The decibel [dB(A)] measurements of traffic noise for the 3 days

The equivalent sound pressure level (L_Aeq_) values were obtained from in situ measurements at nine designated places over three distinct days. As previously stated, the equivalent sound level (Eq. (1)) was calculated at each measurement point from a set of four measurements, each lasting 15 min, resulting in a total of 1 h for each set. Figure 4 (lollipop point plot) presents a descriptive examination of environmental noise levels for the L_Aeq_ indicator. The outcomes of the noise indicator (L_Aeq_) showed that the roadside noise levels (MP 1, 4, and 7) were higher than those at the receptor noise level (MP 2, 3, 5, 6, 8, and 9). The data indicates that L_Aeq_ roadside had a minimum noise level of 61.20 dB(A) and a maximum of 71.80 dB(A), with a mean value of 66.44 ± 2.68 dB(A). Likewise, the L_Aeq_ receptor side had a mean value calculated to be 55.51 ± 3.42 dB, with greater fluctuation, spanning from 49.40 to 64.70 dB(A).

### Validation of the noise maps

The validation of the model was accomplished by comparing the sound levels recorded in the field to those predicted by the model based on traffic volume documented during the research. The model primarily relies on data from traffic counts and road attributes (such as slope and surface type), terrain and elevation data, as well as the number and positions of buildings. The calibration process creates a correction for the computational model, which is either added to or taken away from the calculation result or the noise source’s emission level, depending on the software used, to improve accuracy. If this adjustment is within the allowed limits, then the model and its predictions can be considered valid. The model and its predictions might be considered legitimate if the calibration adjustment falls within the allowed range (meets the predetermined criteria). Nine measurement points on both sides of the barrier yield the equivalent sound levels for both the measuring and model cases.

**Fig. 5:**
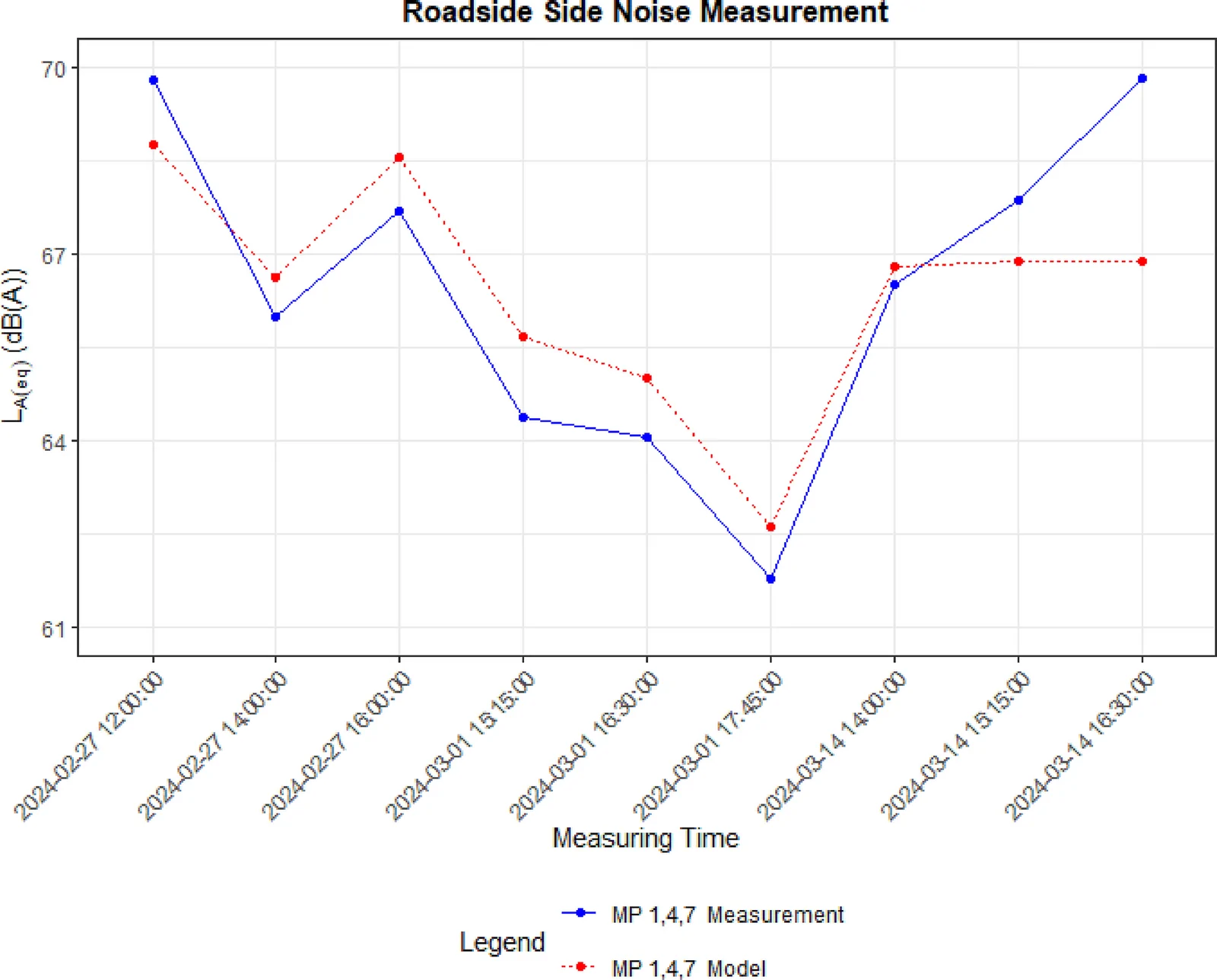
The differences between modelled and measured noise levels along the roadside

**Fig. 6:**
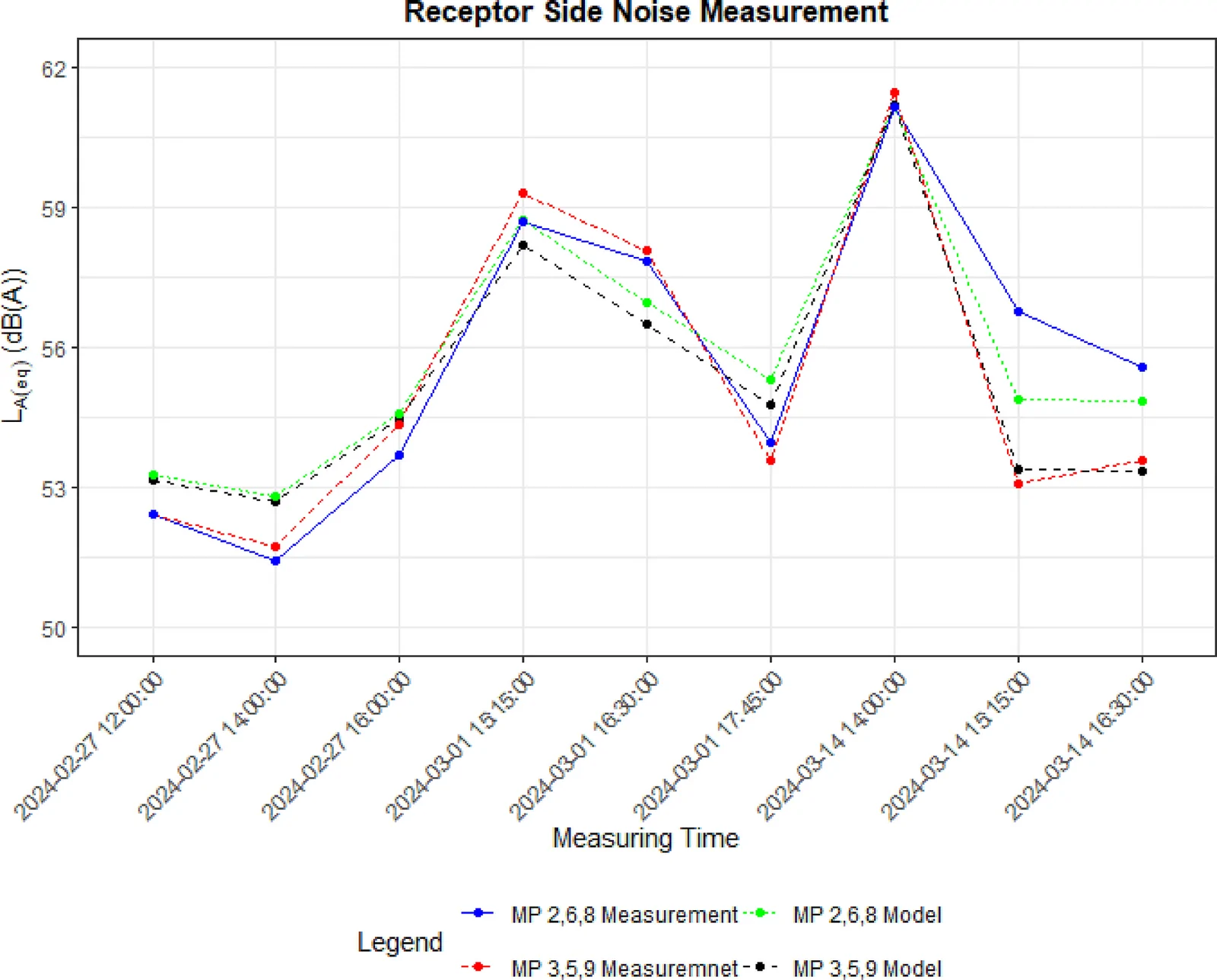
The differences between modelled and measured noise levels along the receptor side

**Fig. 7:**
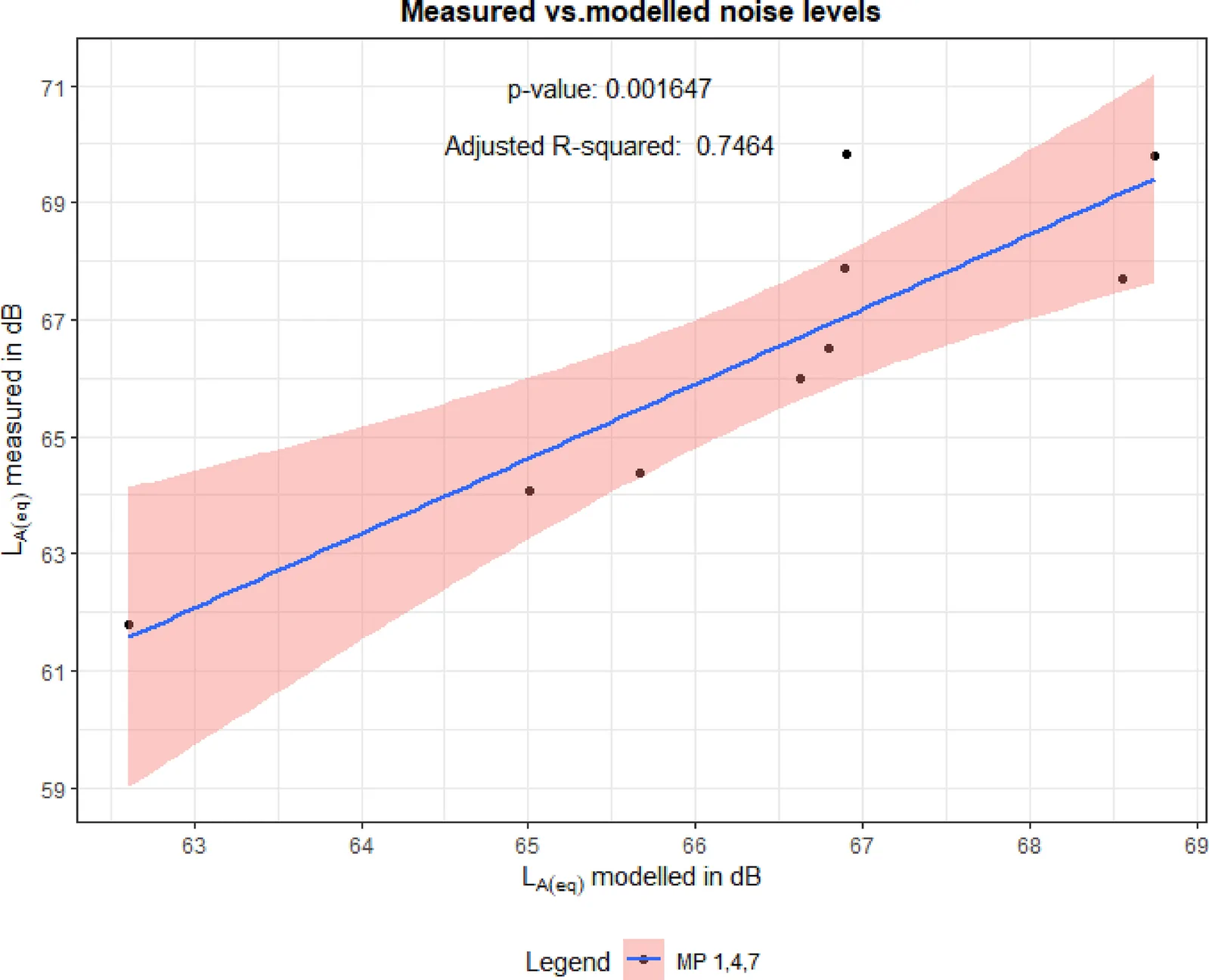
The correlation between the modelled and measured noise levels at the roadside

**Fig. 8:**
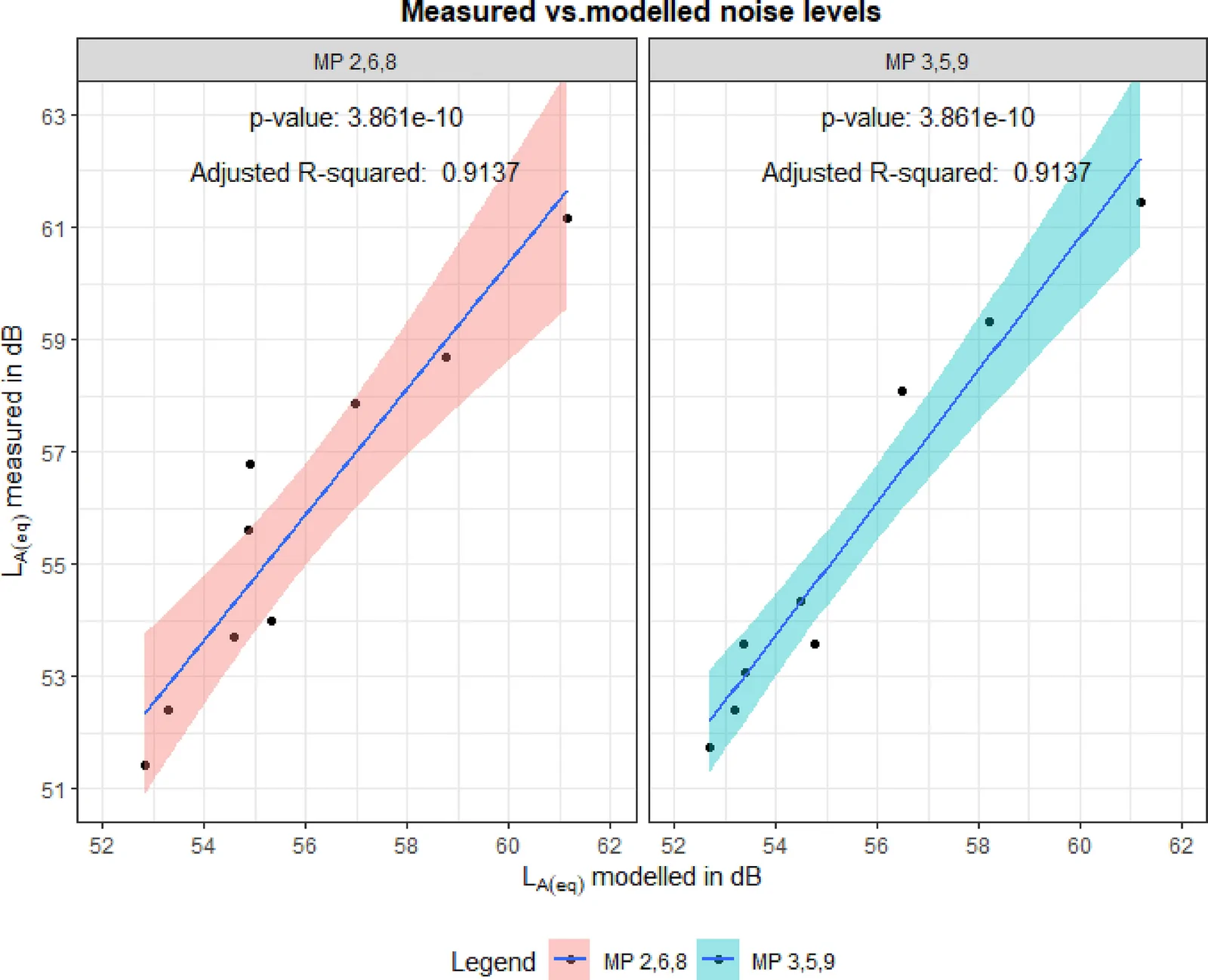
The correlation between the modelled and measured noise levels at the receptor side

Figures 5 and 6 illustrate the results of noise measurements and modelling for both the roadside and receptor sides, respectively. The results indicated that the actual and expected noise levels match closely, with an average validation correction of 1.09 dB(A) on the roadside and 0.81 dB(A) on the receptor side. The results indicated that most of the (L_Aeq_) values calculated using the IMMI software are within the acceptable range of 2 dB(A) (Nast et al., 2014) on both sides of the barrier. Furthermore, research from the US Department of Transportation indicates that road noise modelling outcomes are deemed satisfactory if the uncertainty level is less than 3 dB(A) (Tsai et al., 2019). The correlation coefficients (Figs. 7 and 8) (*r*) were estimated as 0.75 for the roadside and 0.91 for noise values on the receptor side. For the roadside, the L_Aeq_ had a *p*-value of 0.0016. Conversely, for the receptor side, the L_Aeq_ had *p*-values of 3.861e-10, suggesting that both road and receptor L_Aeq_ data follow a normal distribution.

### Noise exposure prediction for future scenario

Noise maps serve as useful tools for controlling ambient noise in cities, as they present a full picture of the issue, including the decibel levels that reach the facades of various buildings. Scenario 0 (reference scenario) is simply based on the actual official traffic data in 2022 for the investigated section provided from ADT data for day and night. After computing the reference noise maps for the current status, a future scenario was generated, aiming to assess the impact of road traffic noise on the population near the Kismacs district after the opening of the new factory, whose workforce will exceed the current population of the district by an order of magnitude. Scenario 1 (future scenario) presents a hypothetical scenario in which there is an increase in the overall number of vehicles on major roadways in 2030, mostly because of the opening of the new factory. All other factors (average speed, road type, etc.) were maintained consistent with scenario 0.

**Fig. 9:**
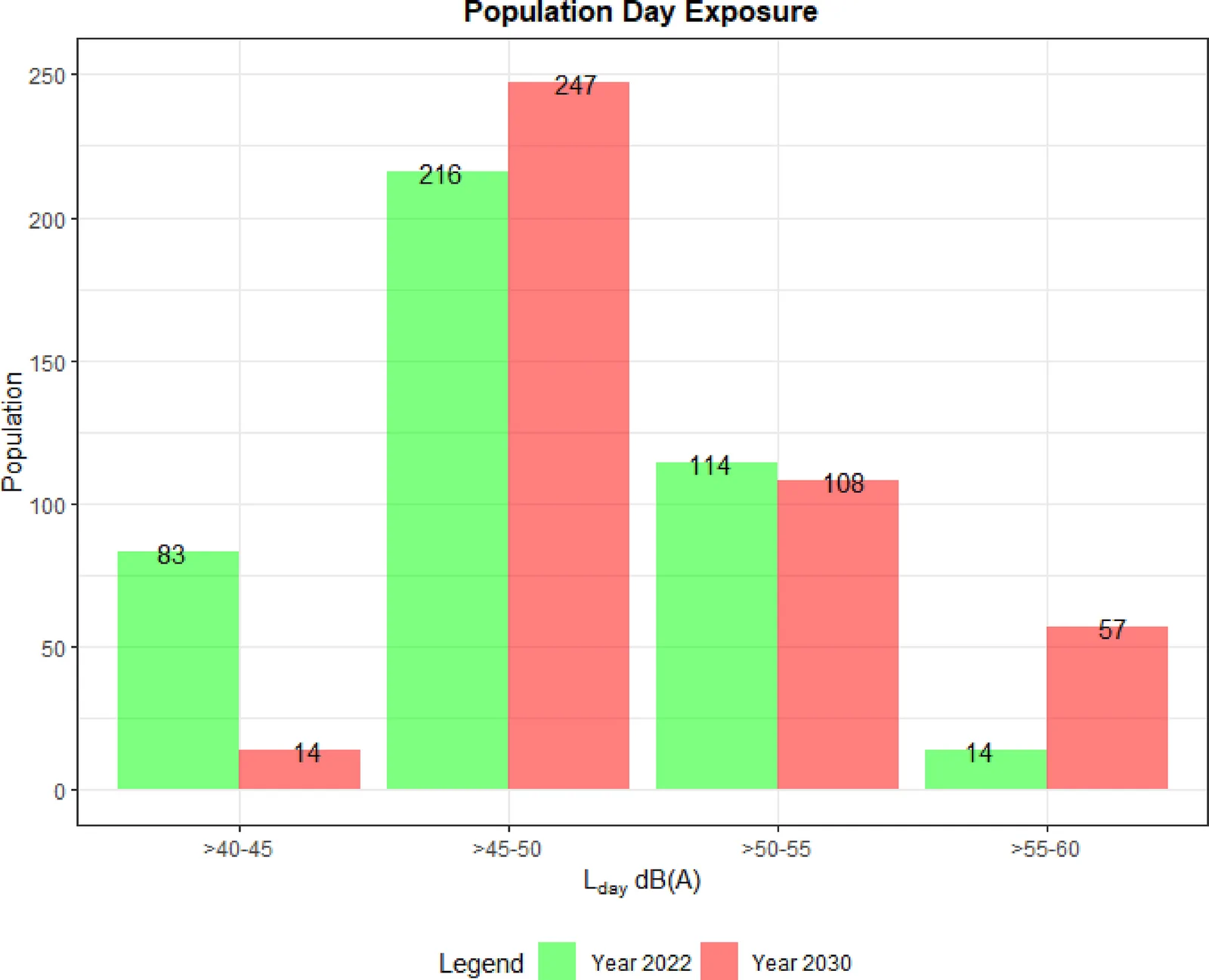
A visual evaluation of population exposure (L_day_) is conducted for the years 2022 and 2030

**Fig. 10:**
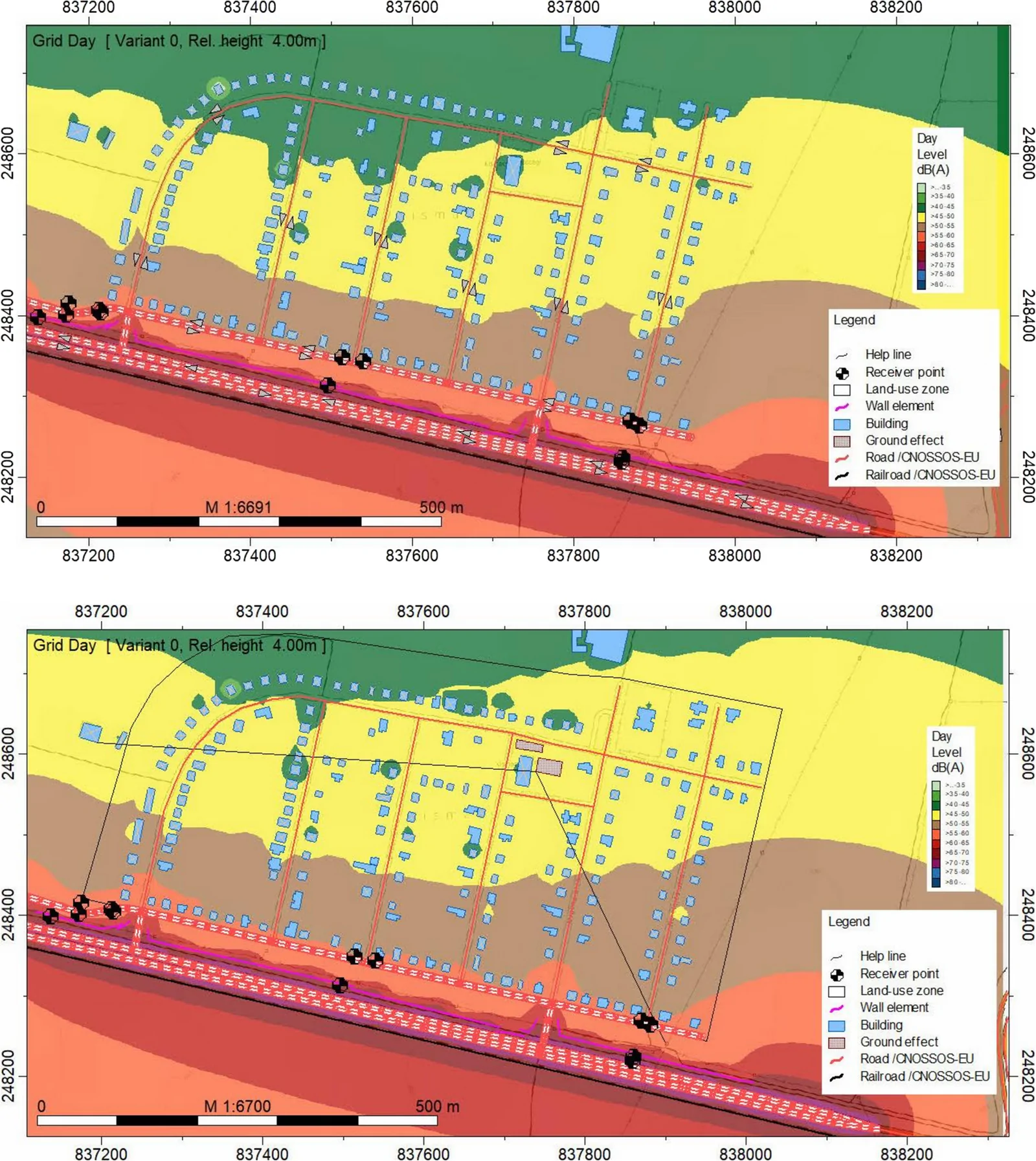
Grid calculation results illustrate the changes in daytime noise exposure at the building façade in the investigated area, contrasting the reference scenario (represented by the top map) with the future scenario (represented by the bottom map)

**Fig. 11:**
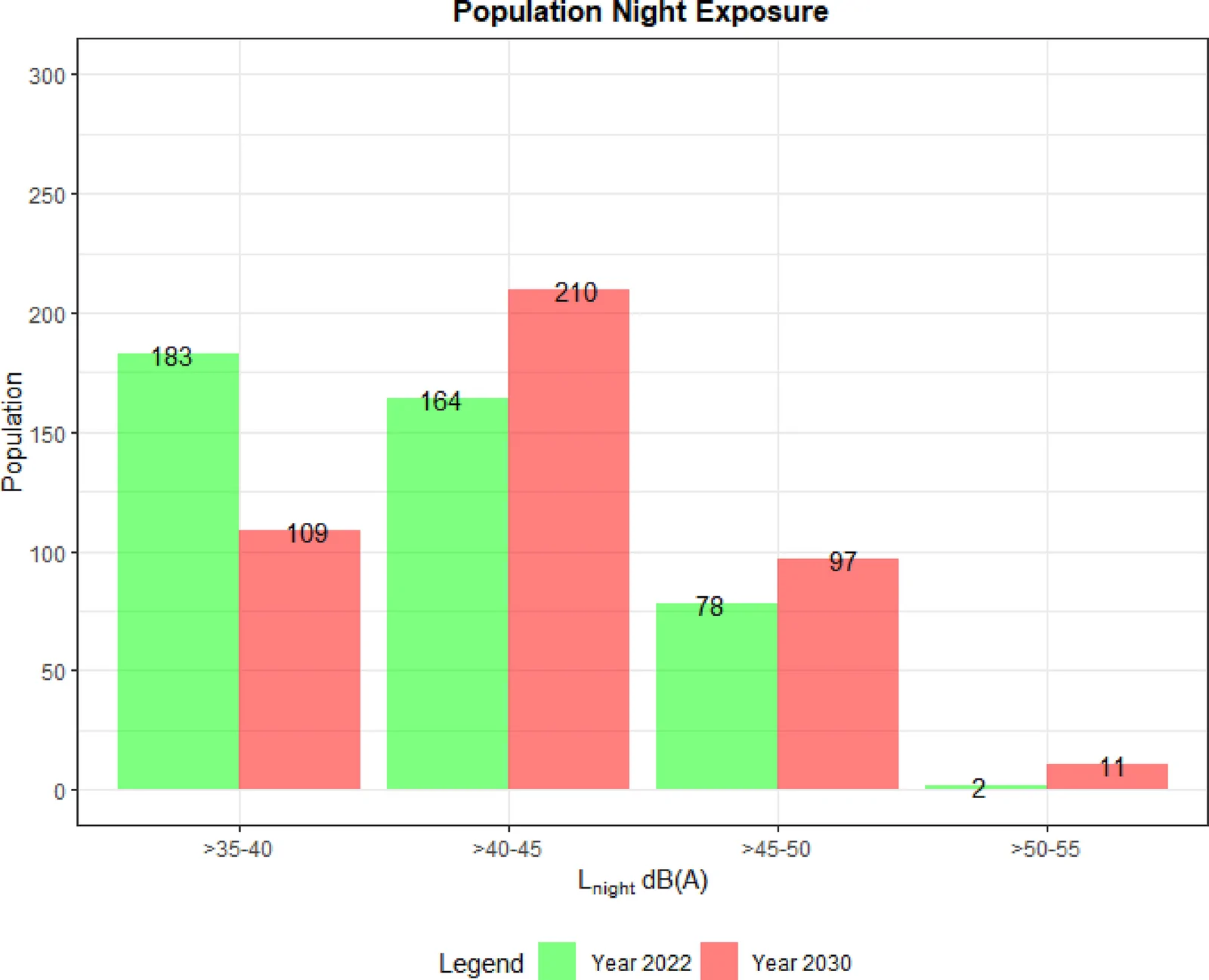
A visual evaluation of population exposure (L_night_) is conducted for the years 2022 and 2030

**Fig. 12:**
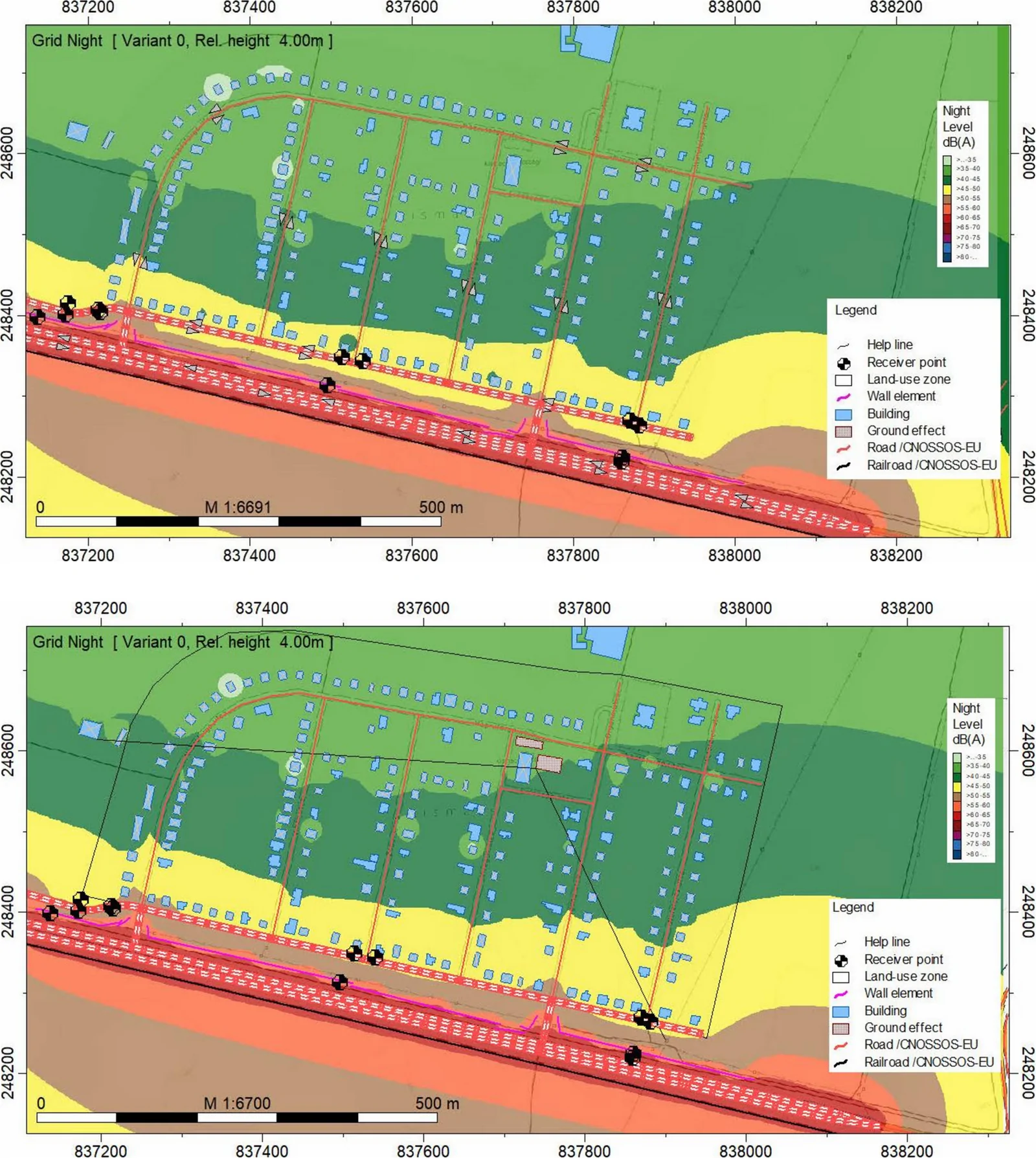
Grid calculation results illustrate the changes in night time noise exposure at the building façade in the investigated area, contrasting the reference scenario (represented by the top map) with the future scenario (represented by the bottom map)

The average hourly traffic (ADT) for 2022 and 2030 is illustrated in Table 1, which entails a 35% increase in overall vehicle traffic on roadways for day and night. The decision was made to highlight some locations where variations in noise levels can be detected. Figures 9 and 10 show the results of the daytime population exposure assessment and noise mapping for the main road studied in this research, comparing the current situation to what is expected in the future. By 2030, it is predicted that there will be a 307.14% increase in the number of people exposed to noise levels between 55 and 60 dB from the road compared to 2022 because of the expected rise in traffic. Figures 11 and 12 show the results for night time, which indicate a 450.00% increase in the number of people who are exposed to sound levels that range between 50 and 55 dB from the roadway in 2030 compared to 2022. However, this finding does not solve the problem of noise pollution in those areas, since the noise levels near the locations are higher than the limits set by the European Environmental Agency; high outdoor noise levels are defined in the 7th EAP as L_den_ over 55 dB and L_night_ over 50 dB (European Environment Agency, 2018). The variations are not substantial, yet they are certainly noticeable. Fewer people are impacted by noise levels exceeding 50 dB, with some classified as “noiseless” in the updated model. Tables 3 and 4 provide a comprehensive summary of the changes.

**Table 3:** Statistical changes in the population's noise exposure in dB(A) for L_day_ in the Kismacs suburban district of Debrecen for 2022 and 2030

L_day_ dB(A)	2022 simulation	%	2030 simulation	%	Difference	Difference %
> 40–45	83	19.44%	14	3.28%	− 69	− 83.13%
> 45–50	216	50.59%	247	57.85%	31	14.35%
> 50–55	114	26.70%	108	25.29%	− 6	− 5.26%
> 55–60	14	3.28%	57	13.35%	43	307.14%

**Table 4:** Statistical changes in the population’s noise exposure in dB(A) for L_night_ in the Kismacs suburban district of Debrecen for 2022 and 2030

L_night_ dB (A)	2022 simulation	%	2030 simulation	%	Difference	Difference %
> 35–40	183	42.86%	109	25.53%	− 74	− 40.44%
> 40–45	164	38.41%	210	49.18%	46	28.05%
> 45–50	78	18.27%	97	22.72%	19	24.36%
> 50–55	2	0.47%	11	2.58%	9	450.00%

As evidenced by Tables 3 and 4, changes in the degree of exposure are noticeable. However, when looking at percentage changes, the most significant differences happen at the quieter noise levels (40–45 dBA L_day_ and 35–40 dBA L_night_), with the number of people exposed to these sounds now decreased by 83.13% and 40.44%, respectively. The findings from noise modelling indicate that changes in traffic volume affect noise levels. The number of individuals and structures that have been newly classified in a higher “noise group” makes these changes distinctly noticeable. Generally, a rise in traffic volume shifts individuals to the higher noise groups. Consequently, the impact of noise on their well-being is more pronounced. Considering the notion of sustainable cities and the significance of each resident, these changes merit implementation.

### Difficulties and limitations

The implementation of the proposed workflow revealed several practical challenges related to data collection, model implementation, and scenario development. The manual vehicle classification was more difficult for fast-moving vehicles and for vehicles travelling simultaneously in different lanes, resulting in a slight overestimation of the heavy vehicle category. Automated traffic counting systems can improve the accuracy of the total traffic counts as well as vehicle classification.

In the absence of actual speed observations, the posted speed limits were used to reflect vehicle speeds. Because of the continuous change owing to acceleration and deceleration, congestion, traffic signals, and driver behaviour, it is difficult to calculate representative speeds. Further, a variation along a whole road segment cannot be captured by a single camera or measuring site. Thus, the posted speed limits were employed as a consistent input for the CNOSSOS-EU model.

The time required to produce a noise map using the proposed workflow is mainly determined by the preparation and quality of the input GIS database rather than by the computational effort of the model itself. Developing a complete spatial dataset, including road network geometry, building footprints and heights, topography, traffic inputs, and receiver locations, is typically the most time-consuming part of the workflow due to data availability and preprocessing requirements. Once prepared, the CNOSSOS-EU calculations implemented in IMMI are computationally efficient, with individual simulation runs typically completed within minutes to several hours depending on study area size and model complexity.

Several practical adaptations were required to implement the methodology in IMMI. In particular, population exposure was estimated using building-based proxies because detailed building-level occupancy information was unavailable. Likewise, standard meteorological assumptions recommended by the CNOSSOS-EU methodology were adopted in the absence of continuous local meteorological observations. These simplifications are common in strategic noise mapping but may introduce additional uncertainty in exposure estimates.

The CNOSSOS-EU methodology provides a robust and harmonised framework for environmental noise assessment, ensuring consistency and comparability across different study areas. Its integration with GIS-based inputs and implementation in IMMI enables efficient and stable large-scale noise calculations. Due to these advantages, it is widely recommended for strategic noise mapping in urban environments requiring transferable and standardised assessment approaches.

Future traffic and environmental circumstances are fundamentally unknown, particularly in rapidly evolving metropolitan environments, where land use, traffic patterns, and infrastructure are in a constant state of change. Defining a single realistic future scenario in such contexts proves challenging, and therefore, one should interpret results as indicative rather than strictly predictive. The quality and temporal relevance of available input data further constrain the reliability of scenario-based modelling. For this reason, practitioners should consider multiple scenario assumptions and regularly updated datasets when applying similar methodologies, rather than relying on a single deterministic forecast. Nevertheless, the proposed workflow provides a useful baseline for noise mapping in comparable, rapidly developing urban areas.

From a practical perspective, future applications of this workflow should prioritise the collection of reliable traffic counts and accurate GIS datasets, as these have the greatest influence on model reliability. Whenever available, automated traffic classification and measured vehicle speeds should be used instead of manual counts and posted speed limits. Population datasets linked to individual buildings should also be incorporated where possible to improve exposure assessment. When feasible, it is advisable to consider multiple traffic growth assumptions in scenario analyses rather than relying on a single deterministic forecast, thereby providing decision-makers with a broader range of possible future outcomes.

## Conclusions

Elevated noise levels in contemporary urban areas have emerged as an environmental concern owing to their detrimental effects on human health. This study used the “CNOSSOS-EU” noise prediction model to evaluate urban road traffic noise by comparing in situ measurements with the model’s results using IMMI noise mapping software. Kismacs, a suburban district of Debrecen, hosted a substantial number of sample points and in situ noise measurements for this purpose. Manual counts were simultaneously conducted on road traffic to determine vehicle statistics, which served as the model's input data. Based on ADT data, current traffic flows were analysed, and a future scenario was generated to predict the effects of anticipated traffic increases on the population in 2030.

The current investigation yielded the following principal conclusions:

- The study calculated the equivalent noise level (L_Aeq_) differences between the noise measurements and modelling results during the “day” time as 1.09 dB(A) for the roadside and 0.81 dB(A) for the noise barrier’s receptor side.
- The noise measurements and the model-calculated results have correlation coefficients (*r*) of 0.75 for the roadside and 0.91 for the receptor side.
- In 2030, a 307.14% increase in the population exposed to daily noise levels between 55 and 60 dB from road traffic is anticipated compared to 2022, due to a 35% increase in average traffic flow.
- In 2030, a 450.00% increase in the population exposed to nightly noise levels between 50 and 55 dB from road traffic is anticipated compared to 2022, due to the same ratio of increase in daily average traffic flow.
- The quantity of people and structures newly categorised inside a higher “noise group” makes these modifications markedly evident.
- Such studies, where real measurement data is used for validation of the noise mapping, can increase its possible utilisation, and they simultaneously increase the efficiency of future predictions.

## Data Availability

Data supporting the study’s findings are available in the paper and Supplementary Information. Any additional data supporting the findings of this study are available upon request.

## Declarations

All authors have read and understood and have complied as applicable with the statement on “Ethical responsibilities of Authors” as found in the Instructions for Authors.
